# Supplementary material for: Consumption of Tritordeum Bread Reduces Immunogenic Gluten Intake without Altering the Gut Microbiota
Source: Foods. 2022 May 16;11(10):1439. doi: 10.3390/foods11101439 (PMC9142130; doi:10.3390/foods11101439)
Supplement: Supplementary file 1 [file foods-11-01439-s001.zip › Figure S1.pdf]

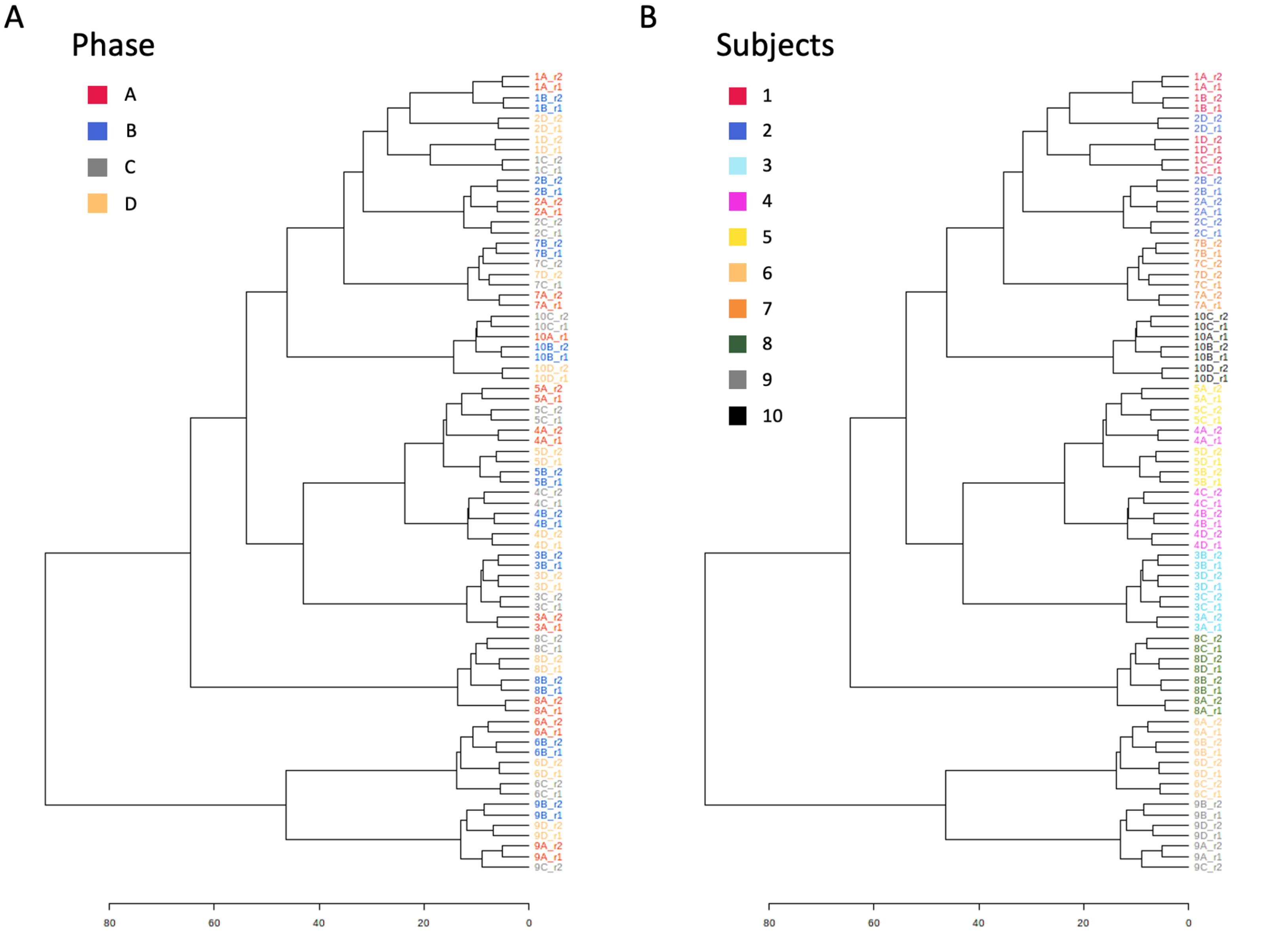

Supplementary Figure S1. Hierarchical cluster analysis dendrogram using the Euclidean distance and Ward method as clustering algorithm. (A) The samples are colored according to the phase of study to which they belong. (B) The samples are colored according to the subject to which they belong. The samples are coded as follows: the first number corresponds to the subject, the letter corresponds to the study phase, and r1 or r2 corresponds to replica 1 or 2, respectively.
